# A Degeneration Gradient of Poplar Trees Contributes to the Taxonomic, Functional, and Resistome Diversity of Bacterial Communities in Rhizosphere Soils

**DOI:** 10.3390/ijms22073438

**Published:** 2021-03-26

**Authors:** Juan Liu, Xiangwei He, Jingya Sun, Yuchao Ma

**Affiliations:** 1 Department of Microbiology, College of Biological Sciences and Biotechnology, Beijing Forestry University, Beijing 100083, China; cfb0405@163.com (J.L.); hexiangwei@bjfu.edu.cn (X.H.); sunjingya0303@163.com (J.S.); 2 Beijing Advanced Innovation Center for Tree Breeding by Molecular Design, Beijing Forestry University, Beijing 100083, China

**Keywords:** the degeneration of poplar, metagenomic analysis, microbial communities, metabolic pathway, resistance genome

## Abstract

Bacterial communities associated with roots influence the health and nutrition of the host plant. However, the microbiome discrepancy are not well understood under different healthy conditions. Here, we tested the hypothesis that rhizosphere soil microbial diversity and function varies along a degeneration gradient of poplar, with a focus on plant growth promoting bacteria (PGPB) and antibiotic resistance genes. Comprehensive metagenomic analysis including taxonomic investigation, functional detection, and ARG (antibiotics resistance genes) annotation revealed that available potassium (AK) was correlated with microbial diversity and function. We proposed several microbes, *Bradyrhizobium*, *Sphingomonas*, *Mesorhizobium*, *Nocardioides*, *Variovorax*, *Gemmatimonadetes*, *Rhizobacter*, *Pedosphaera*, *Candidatus Solibacter*, *Acidobacterium*, and *Phenylobacterium*, as candidates to reflect the soil fertility and the plant health. The highest abundance of multidrug resistance genes and the four mainly microbial resistance mechanisms (antibiotic efflux, antibiotic target protection, antibiotic target alteration, and antibiotic target replacement) in healthy poplar rhizosphere, corroborated the relationship between soil fertility and microbial activity. This result suggested that healthy rhizosphere soil harbored microbes with a higher capacity and had more complex microbial interaction network to promote plant growing and reduce intracellular levels of antibiotics. Our findings suggested a correlation between the plant degeneration gradient and bacterial communities, and provided insight into the role of high-turnover microbial communities as well as potential PGPB as real-time indicators of forestry soil quality, and demonstrated the inner interaction contributed by the bacterial communities.

## 1. Introduction

The rhizosphere soil influenced by root secretions contains up to one hundred billion microbial cells per gram root [1] and about thirty thousand prokaryotic species [2]. As the second genome of the plant, the complex root-associated microbial community is crucial for nutrition and plant health [3]. A variety of organic compounds including carbohydrate, organic acids, phenol, and other substances secreted by plant roots in the surrounding soil, which are the sources of nutrients and vitamins for various bacteria, stimulating the development of soil microbial communities and changing their activity and ecological distribution [2,4]. Microbes are in competition for nutrients and space with many other microbes in the rhizosphere to affect them to a certain extent through a complex network of interactions. The beneficial microbiome associated with roots, including the so-called plant growth promoting (PGP) bacteria, can contribute to alleviate plant stress by a variety of mechanisms [5]. Among them, PGP bacteria can directly enhance micronutrient uptake and affect phytohormone homeostasis, or indirectly stimulate the plant immune system against phytopathogens [6] and improve soil texture and structure [5,7]. Recent evidence suggests that differences among plant species when grown on the same soil or between plant genotypes have a significant impact on the rhizosphere microbiome [8,9]. However, we were unable to formulate how root microbiota change under different plant phenotype, especially artificial vegetation under natural conditions.

As a woody perennial organism, *Populus* has become the model for insight into the mechanistic hypotheses related to plant–microbe interactions because of its fast growth rates and full genome sequenced. Several studies have demonstrated that microbial isolates from poplars can enhance the health, growth, development and resistance to biotic and abiotic stresses of their plant hosts [10,11,12]. The core microbiome and niche-level microbiome of poplar were dissected and archaeal/bacterial diversity from rhizosphere soil was richer than that in leaf, stem and root regardless of plant genotype [13]. The composition of poplar rhizosphere microbiome was significantly different across environmental gradients or genotypes [14,15]. However, little is known that the assembly and shift of the microbial consortia within or between *Populus* phenotypes.

*Populus* are not only used in paper industry, as a cellulose derived fuel raw materials, but also usually in artificial afforestation to protect soil erosion. Restoration of vegetation is undoubtedly an important measure to reduce air pollution, prevent wind and fix sand, and cope with desertification and global warming [16,17,18]. Many countries and regions have carried out natural forest protection and artificial forest cultivation programs [19], among which the Three North Shelterbelt known as “Green Wall of China” as one of the typical examples. During the decades of the construction of the Three-North Shelterbelt, many studies have made evaluations based on its achievements, and the extensive ecological benefits obtained are beyond doubt [20,21,22]. However, in recent years, with the impact of climate change and human activities, large scale *Populus* trees degeneration with dieback symptom and partial aggravating trend have been observed in some areas of the artificial forest [23]. Especially in Zhangbei County of Hebei Province, nearly one third of the *Populus* trees, as the main species in the local artificial forests, exhibit rapid widespread degeneration [24].

**Figure 1:**
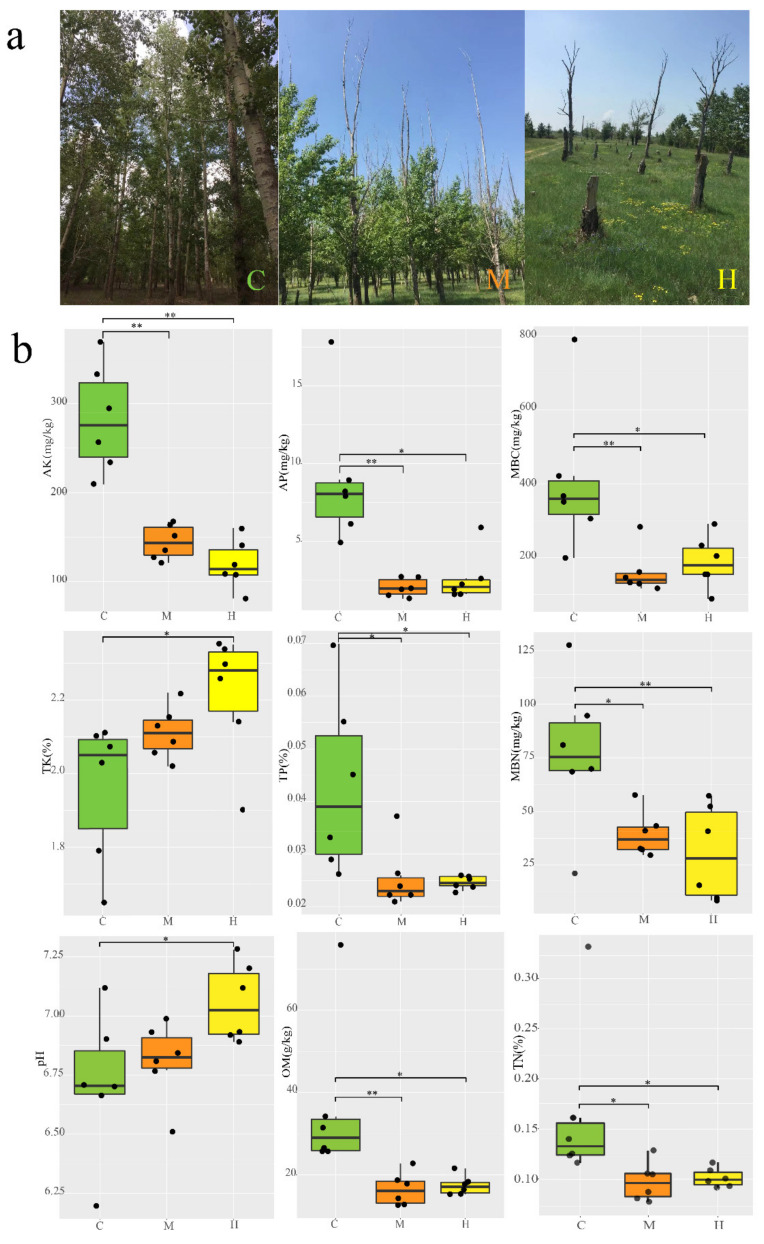
Poplar tree growth conditions at the sampling site and comparison of physical and chemical factors between groups. (**a**) Actual diagram of different growth states of poplar. Poplars in undegraded condition (C); Medium-degraded poplars from the treetop (M); Completely degraded poplars (H); (**b**) variation in soil properties between different groups, with different parallel samples in the group set as the black dots. ** *p <* 0.01, * *p <* 0.05 (After Shapiro-Wilk and Levene’s test, Anova with LSD method or Kruskal–Wallis tests and multiple comparison with Bonferroni method were carried out.).

Many studies have been put forward to explain the natural factors caused the degradation of the Three North Shelterbelt from the aspects of afforestation methods, soil physical and chemical properties, climate and environmental conditions and tree physiology [19,25,26,27]. Changes in temperature and precipitation exacerbate the frequency and intensity of drought, as well as the timing mismatch between resource demand and local and regional resource supply, increasing plant stress and mortality according to the survey of artificial forests [25,26]. Poplar bacterial canker disease and insufficient soil nutrients were also associated with degeneration and mortality [27,28,29,30]. However, few researches have directly investigated the genetic profile of poplar root microbial community, and there is a knowledge gap in understanding taxonomic, functional, and resistome diversity of rhizosphere microorganisms of poplar under different health conditions. Here, we hypothesized that rhizosphere soil microbial diversity and function varies along a degeneration gradient of poplar. In this study, based on Illumina PE150 metagenome sequencing platform, the microbial community structure, functional, and resistance genes of *Populus* rhizosphere soils were comparatively analyzed for undegenerated, medium-degenerated, and completely degenerated *Populus*, respectively (Figure 1a). These results in comparative analysis of the microbiome composition will lay a foundation for further revealing the interaction mechanism between *Populus* and its rhizosphere microorganisms, and also provide a theoretical basis for rational cultivation of *Populus* as an ecological tree species.

## 2. Results and Discussion

### 2.1. Populus and Rhizosphere Soil Characterization

Three representative plots (C, M and H) were defined based on the different degeneration degrees and the growth characterization of *Populus* were carried out (Figure 1a). The tree features of H plot were not detected, because the dead trees were recognized to have been cut down and cleared by local authorities, leaving only the stumps. The average diameter at breast height (DBH) and tree height of poplar trees were 15.46 cm and 21.49 m in C plot, but only 11.56 and 18.30 m in M plots, respectively. In M plot, more than 92% of the trees experienced treetop dryness to varying degrees, and the average height of the dry part was 6.82 m. The overall canopy density of C was 0.83, that of M was only 0.54 (Table S1) because of the dieback of the trees. In addition, five-point sampling (1 m × 1 m) was carried out using the diagonal principle in the C, M, and H plots to understand the species richness in each plot (Table S2). The average species coverage of C, M, and H was 17.2%, 71.8%, and 79.4%, respectively. As treetops dry up, a lower canopy density in medium-degraded plot to receive more sunlight, resulting in richer plant species.

Previous research suggested that soil characteristics not only could reflect soil productivity, but also played important roles in shaping the soil bacterial community diversity and structure in the terrestrial ecosystem [31]. Trees healthy had a strong significant effect on all the soil physical and chemical properties. Nine soil chemical characteristics were selected and measured to compare the differences among C, M, and H group (Figure 1b). The content of total nitrogen (TN), total phosphorus (TP), microbial biomass carbon (MBC), microbial biomass nitrogen (MBN), available phosphorus (AP), available potassium (AK), and organic matter (OM) were highest in sample C with 0.166%, 0.143%, 405.7 mg/kg, 77.2 mg/kg, 8.97 mg/kg, 282.94 mg/kg, 36.62 g/kg and markedly (*p* < 0.05 or *p* < 0.01) higher than in sample H, which were similar between H and M soils. All the pH were close to neutral, but presented a differing variation: H > M > C samples (rough trend) with 6.72, 6.81, and 7.06, respectively. It is worth noting that the content of total potassium (TK) was highest with 2.21%, but AK was lowest with 119.7 mg/kg in H, respectively. In summary, the soil characteristics with different *Populus* growth conditions in the same area exhibited obvious differences, especially between C and H plus M. The soil fertility was on full displayed by the physiological and biochemical characteristics, which perhaps lead to the growth distinction of the *Populus*.

### 2.2. Sequencing Results

The 18 soil samples subjected to metagenomic sequencing generated more than 101 Gb of raw data (average of 5.6 Gb per sample). After quality control and removal of poplarsequence (host contamination 0.663–0.06%), filtered sequencing data per sample reduced to 97.35% of the average raw data. The assembly of the reads using MEGAHIT software generated an average of 636,794, 573,556 and 504,433 scaftigs in C, M, and H, respectively (Table S3). The lengths of scaftigs N50 were between 646 bp to 850 bp, and those of scaftigs N90 were between 520 bp to 539 bp. These scaftigs (≥500 bp) were clustered into gene catalogs (unigenes) after ORF (Open Reading Frame) prediction and filtering (≥100 nt). 9,660,445 non-redundant ORFs were generated from metagenomic libraries, of which 0.21 million (15.1%) were complete ORFs containing both starting and ending codons. 49.89% of the unigenes can be assigned to taxonomic annotations, which suggesting that there was a huge resource pool for novel functional gene in rhizosphere soil of the shelterbelt poplar. About 99.86% of the total annotated unigenes were prokaryotic (bacteria and archaea). Only 0.06% and 0.01% of the unigenes were annotated as eukaryotic (including fungi, protozoa, algae, and plants) and virus, respectively, indicating that the eukaryotic and viral communities have been seriously underestimated due to potential annotation bias. Several studies found generally high levels of fungal diversity in association with transgenic poplar roots [32,33,34]. However, in this study, eukaryotic abundance was only 0.03%, indicating a unique feature of microbial community in poplar rhizosphere of shelter forest.

The percentage of bacterial was significantly higher in samples from C than those from M and H (*p* = 0.0012, *p* = 0.00023), whereas the proportion of archaea increased from sample C to sample H (*p* = 0.012, *p* = 0.0011). Three type samples shared 5,530,921 genes, and 161,741 and 108,437 genes were found exclusively in samples H and M, respectively. By contrast, the sample C contained about six times as many peculiar genes as the other two samples (Figure S1a), which suggested that the sample C is annotated to a richer microbial community. The correlation analysis among different samples revealed six parallel samples within each group have higher similarity, and distinct differences between types (Figure S1b).

### 2.3. Comparative Analysis of Microbial Communities

**Figure 2:**
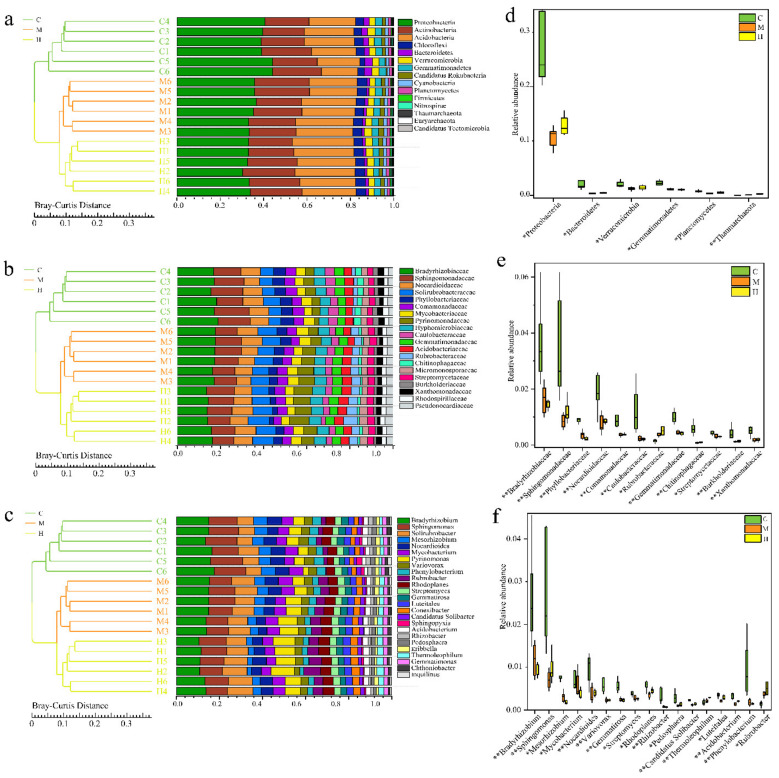
Comparative analysis of bacterial communities at different taxonomic levels. (**a**–**c**) Unweighted pair-group method with arithmetic mean (UPGMA) clustering based on Bray–Curtis distances and the relative abundance of the most abundant bacteria at the phylum, family, and genus level; (**d**–**f**) Indicator bacteria with significant differences between C, M, and H groups. “*” represents adjust *q* value < 0.05, “**” represents adjust *q* value < 0.01.

Bray–Curtis distance matrix was used for sample clustering analysis to construct the total prokaryotic profile among samples at different taxonomic levels from phylum to genus. A total of 160 phyla were recovered from all samples. The most abundant bacteria phyla were *Proteobacteria* (36.75% ± 6.85%), *Acidobacteria* (22.2% ± 5.7%), and *Actinobacteria* (21.9% ± 2.9%) (Figure 2a). The five most abundant prokaryotic families were *Sphinogomonadaceae*, *Bradyrhizobiaceae*, *Phyllobacteriaceae*, *Solirubrobacteraceae,* and *Pyrinomonadaceae*, which comprised on average 33.24% of the bacteria communities (Figure 2b). At the genus level, *Bradyrhizobium*, *Sphingomonas,* and *Solirubrobacter* were the most three abundant across all communities (Figure 2c).

By comparing the relative abundance of prokaryotes among the three sampling sites on the above-mentioned taxonomic level, revealed the great difference on microbial communities between C and H groups. Among the three sample groups, *Proteobacteria* is the most abundant of all the phylum showing significant differences, and the abundance from C to M and H significantly decreased (*q* = 0.00018, *q* = 0.0042, Hypothesis Testing), but there was no significant difference between M and H (Figure 2d). The main families, including *Bradyrhizobiaceae*, *Sphingomonadaceae*, *Phyllobacteriaceae*, *Nocardioidaceae*, *Comamonadaceae*, *Caulobacteraceae*, *Gemmatimonadaceae*, *Chitinophagaceae*, *Burkholderiaceae*, and *Xanthomonadaceae*, and main genera, including *Bradyrhizobium*, *Sphingomonas*, *Mesorhizobium*, *Nocardioides*, *Variovorax*, *Gemmatimonadetes*, *Rhizobacter*, *Pedosphaera*, *Candidatus Solibacter*, *Acidobacterium*, and *Phenylobacterium*, showed the similar trend (Figure 2e,f). Moreover, *Mesouhizobium*, *Rhodoplanes*, *Luteitalea,* and *Streptomyces* were also the microbes with obviously higher abundance in C than in H groups (*q* < 0.05), which were similar in H and M groups. In addition, the abundance of *Thermoleophilum* at phylum level, *Rubrobacteraceae* at family level, *Thermoleophilum* and *Rubrobacter* at genus level decreased from H to C (*q* < 0.01).

In terms of prokaryotes, *Proteobacteria*, *Actinobacteria* and *Acidobacteria* are the dominant phyla, as well as the rhizosphere of many other hosts [35,36]. At the genus level, *Bradyrhizobium* and *Mesorhizobium* contains species widely known as nitrogen-fixing bacteria with legumes, and they have an outstanding ability to promote the growth of non-legumes through the production of indole-3-acetic acid (IAA) and siderophores, the solubilization of potassium and phosphate [37]. Members of the *Sphingomonas* genus increased *Arabidopsis thaliana* growth rate, alter rhizosphere microbial community structure of plant under drought stress [38], and alleviate Cd stress in oilseed rape through regulation of the GSH-AsA cycle and antioxidative enzymes [39]. *Variovorax* genus has the potential to promote plant growth, due to its ability to hydrolyze cellulose [40] and to produce plant growth enzymes and substances such as siderophores, IAA and 1-aminocyclopropane-1-carboxylic acid deaminase [41,42,43]. The high relative abundance of *Verrucomicrobia* was found in grassland soils of a coniferous forest [44], in the rhizosphere of *Arabidopsis thaliana* [45], poplar [46] and maize [47,48], and in rice roots [49,50,51]. *Verrucomicrobia* has been evidenced that play critical roles in environmental carbon cycling and (poly) saccharide degradation because of high coding densities for glycoside hydrolase genes in the genome [52,53]. Memebers of *Verrucomicrobia* colonized in intercellular spaces and inside root cells of rice, and produced IAA and its precursors IAM (indole-acetamide) or IPA (indole-3-pyruvic acid) for the plant growth promotion, showed activity of acid phosphatases for the solubilization of organic phosphates, and possessed also genes for citrate synthase [45]. Members of the *Sphingomonas* and *Variovorax* genera can survive in low nutrient environment, often be isolated from oil-contaminated soil due to their unique wide range of xenobiotic-biodegradative abilities [54,55]. *Streptomyces* bacteria, famous for producing secondary metabolites including antibiotics, may be used as plant probiotics due to the beneficial effects on plants and are not only ubiquitous in soil, but also are isolated from plant roots [56]. In summary, multiple members enriched genera in the healthy poplar rhizosphere are well known as beneficial plant microorganisms, which can maintain the hormonal balance, promote the acquisition of nutrients and development of the root system in its plant host, and thus prevent plant diseases.

It is worth noting that only two genera *Thermoleophilum* and *Rubrobacter* have higher content in H than C. *Thermoleophilum*, *Rubrobacter* are thermophilic and radiation-resistant strains [57], which may be the reason why they can survive in a relatively poor rhizosphere environment. Studies have shown that the same plant host can recruit similar microorganisms to form a rhizosphere environment on different soils [58]. It can be seen that there is little significant difference in microbial diversity among all the samples, and dominant microbes are widely found in nature. However, compared with H and M samples, C group has a more abundant microbiome. This may indicate a denser material exchange between poplars rhizosphere and host in C group. Importantly, how to link them with drought resistance and disease resistance.

### 2.4. Correlation Analysis of Soil Characterization and Microbial Communities

**Figure 3:**
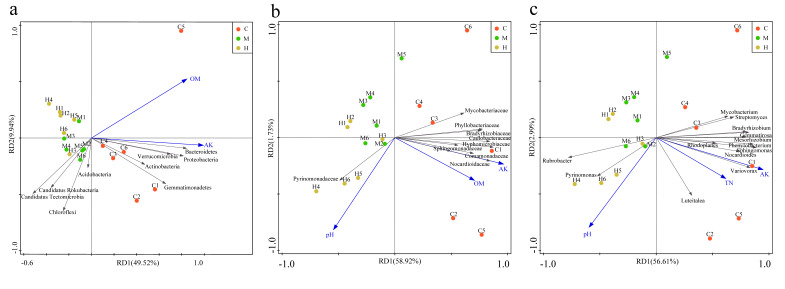
Redundancy analysis for the microbes among samples at (**a**) phylum, (**b**) family, and (**c**) genus levels with edaphic physicochemical factors, respectively.

Within a host species, habitat and soil type, rather than host genetic background, have larger effects on the overall structure of the microbiome, but the balance of the effects of genetic and soil factors within host habitats on bacteria is less clear [15,47,59,60]. According to principal component analysis (PCA) and the redundancy analysis (RDA) based on the physicochemical properties of the soil and the most abundant prokaryotes among the samples, the three groups were separated obviously at the level of phylum, family and genus. At the level of phylum, *Bacteroidetes*, *Proteobacteria*, *Verrucomicrobia*, *Actinobacteria,* and *Gemmatinonadetes* were significantly positively correlated with OM and AK, while negatively correlated with *Candidatus Tectomicrobia* and *Candidatus Rokubacteria* (*p* < 0.05) (Figure 3a). At the family level, *Sphingomonadaceae*, *Comamonadaceae*, *Nocardioidaceae*, *Hyphomicrobiaceae*, *Caulobacteraceae*, *Bradyrhizobiaceae*, *Phyllobacteriaceae,* and *Mycobacteriaceae* were significant positive correlation with AK and OM (Figure 3b). At genus level, *Nocardioides*, *Variovorax*, *Sphingomonas*, *Phenylobacterium*, *Rhodoplanes*, *Mesorhizobium*, *Bradyrhizobium*, *Streptomyces*, *Mycobacterium,* and *Luteitalea* were positive correlated with AK and TN (Figure 3c). *Pyrinomonadaceae* at family level, and *Rubrobacter* and *Pyrinomonas* at genus level were significant positive correlation with pH (Figure 3b,c). Almost all the members positively associated with AK, OM, and TN were enriched in group C, but the members positively associated with pH were enriched in H, which suggested that pH, AK, OM, and TN were the indicators to identify the poplar rhizosphere soil fertility. In particular, AK was the best indicator to distinguish community distribution of group C from the other two sample sat three taxonomic levels (Figure 3).

Potassium is the third essential nutrient factor of plants, which plays a key role during growth, development and metabolism of plants. If potassium supply is insufficient, plants will be more susceptive to diseases and pest with slow-growing, small seeds, poorly developed roots, and lower yields [61,62,63]. Potassium-soluble bacteria in soil can convert insoluble or mineral potassium compounds into soluble forms for use by plants as soil solutions [64]. A variety of organic acids produced by potassium-solubilizing bacteria, such as tartaric acid, lactic, acetic, propionic, citric, oxalic, glycolic, succinic acid, fumaric, malonic and tartaric, mobilized and solubilized the insoluble potassium and structural unavailable forms of potassium compounds [65]. The maintenance of soil quality is a key factor for environmental sustainability, while the increase of microbial diversity with various functions, such as organic matter decomposition, potassium, and phosphorus solubilization, and nitrogen fixation etc., significantly affecting soil quality and sustainability [66]. Our results suggest that changes in bacterial community composition and diversity might be related to the AK influenced by the shifts in biogeochemical processes, which generally limit plants growth and fitness.

### 2.5. Comparison of Microbial Interaction Networks

**Figure 4:**
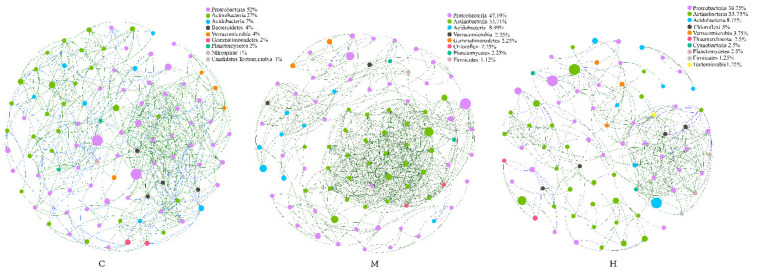
Community networks of C, M and H set up co-occurrence based on top 100 genera respectively. Each node represents a genus, and the size and color represent its abundance and the annotation color of the phylum which it belongs, respectively. Green line—positive correlation, blue line—negative correlation.

To identify potential microbe–microbe interactions in poplar rhizosphere under different health conditions, we construct three bacterial co-occurrence networks (Figure 4). The network topological metrics showed that microbial co-occurrence patterns were markedly different among C, M, and H. The average degree (avgK) and network density values (Table S4) were similarity in C and H, but higher than that in M. Compared with the H group, the co-occurrence network established from C and M samples was more compact and complex, which was intuitively shown in the Figure 4. The network of group C was more homogeneous, while positive links in group M were significantly increased, which may be due to the dense positive correlation of genera belonging to *Actinobacteria* with each other. These observations suggested that more intimate host-microbe and microbe–microbe interactions occur in the healthy poplar rhizosphere than in the degradated poplar rhizosphere.

Although low abundance genera may contribute significantly to the function of the whole community, genera with high abundance and more connectivity links obviously play a critical role in the structural stability of the entire microbial network, considered as key species. *Sphingomonas* belong to *Proteobacteria*, *Rubrobacter* and *Solirubrobacter* belong to *Actinobacteria*, *Pyrinomonas* belong to *Acidobacteria* were found to be the key genus of Group C, M, and H, respectively.

### 2.6. Function Potential Analysis of Microbes

Functional annotations showed approximately 43% of the ORFs were assigned to the KOs by blasting against the KEGG Orthology (KO) database. At the first level of KEGG targeted the metabolism as the main functional pathway (54.24%), followed by genetic information processing (15.36%), environmental information processing (11.78%), cellular processes (9.41%), organismal systems (5.65%), and the human diseases (3.55%) (Figure S2). The 7108 KOs were mainly involved in 405 KEGG pathway at level 3 in total and the top 35 pathways accounted for about 56% of all metabolic pathways.

**Figure 5:**
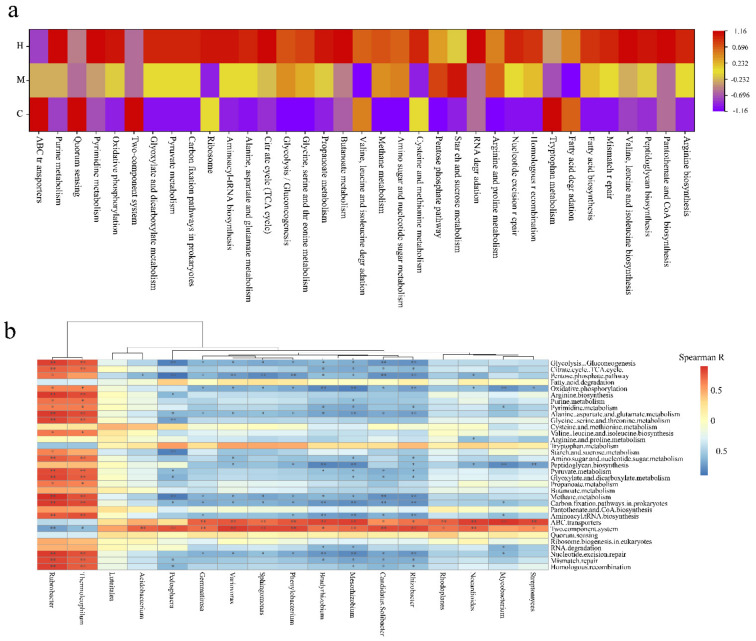
(**a**) Heat map analysis of the top 35 most abundant Kyoto Encyclopedia of Genes and Genomes (KEGG) pathways in at least one soil sample using normalized abundance. Heat map is color-coded based on row z-scores. (**b**) Spearman correlation analysis between genus with significant difference between C and H and metabolic pathway on level 3. “*” represents *q* value < 0.05; “**” represents *q* value < 0.01.

Plant–microbe and microbe–microbe interactions are very likely to be important factors that would influence the assembly of rhizosphere microbiomes. The samples C and H were enriched and compared in different metabolic pathways (Figure 5a). The KOs involved in known plant–microbe and microbe–microbe interactions, such as quorum sensing and two-component system, were over-represented in C (Figure 5a). These observations suggested that more intimate host-microbe and microbe–microbe interactions occur in the healthy poplar rhizosphere than in the degradated rhizosphere. Tryptophan is an important precursor of auxin biosynthesis. Tryptophan metabolism was enriched in C, indicating that the bacteria in healthy poplar rhizosphere had outstanding ability to promote plant growth. Nutrition in the rhizosphere and surrounding environment of host plants plays a key role in the construction and stabilization of the microbiome. Plant-derived compounds in the rhizosphere soil would be likely nutrient sources for microbes. Consistent with this, ABC transporters responsible for transporting plant-derived nutrients, such as peptide, amino acid, vitamin B12, monosaccharide, oligosaccharide, polysaccharide, lipid, mineral, organic ion, and iron-siderophore, into microbial cells, were over-represented in C group. Degradation of poplar trees may result in reduced secretions and more difficulty for microorganisms to obtain nutrients. Consistent with this, the KOs that include carbon metabolism, synthesis of coenzymes, and nucleic acid metabolism, such as, oxidative phosphorylation, glyoxylate and dicarboxylate metabolism, carbon fixation pathways in prokaryotes, pyruvate metabolism, citrate cycle (TCA cycle), glycolysis/gluconeogenesis, propanoate metabolism, pantothenate and CoA biosynthesis, butanoate metabolism, purine metabolism, and pyrimidine metabolism, were over-represented in H. Such findings were consistent with the fact that healthy poplar rhizosphere microorganisms could acquire a variety of simple nitrogen and carbon sources from root exudation, and therefore would not need to invest in their biosynthesis. The enrichment of pathways involved in the amino acid synthesis and metabolism, such as valine, leucine, isoleucine, arginine, aminoacyl-tRNA biosynthesis, and ribosome, alanine, aspartate, glutamate, glycine, serine, threonine, cysteine, and methionine metabolism, was observed in H. The amino acids recycling depleted in C further suggested that rhizosphere microbes can directly obtain amino acid from root exudates, and that amino acids represent an important nitrogen and/or carbon source. In addition, the KOs involved in adverse variation repair, such as RNA degradation, nucleotide excision repair, homologous recombination and mismatch repair, were enriched in H. These repair pathways and RNA degradation are important ways for cell to respond to environmental changes.

Subsequently, the Spearman correlation coefficient was calculated to reveal the contribution of microbiome to metabolic pathways based on the 17 most abundant genera and pathway profiles with significant differences between groups (Figure 5b). The results show the following multiple important genera, *Gemmatirosa*, *Variovorax*, *Sphingomonas*, *Phenylobacterium*, *Bradyrhizobium*, *Mesorhizobium*, *Candidatus Solibacter*, *Rhizobacter*, *Rhodoplanes*, *Nocardioides*, *Mycobacterium*, and *Streptomyces*, were main driving factors of ABC transporters and two component system. In addition, *Acidobacterium* and *Pedosphaera* also drove two component system. Most of above genera, were enriched in large quantities, and ABC transporters and two-component systems were also enriched in C, which indicate that the host–microbe and microbe–microbe interactions were more intimate in healthy poplar rhizosphere than in degradation poplar rhizosphere. The KOs involved in carbon and energy metabolism, amino acid cycling, nucleic acid repair, and homologous recombination were driven by *Rubrobacter* and *Thermoleophilum* belong to *Actinobacteria*. These KOs and the two genera were enriched in the H, which indicated that microbes required stronger basal metabolism to survive with a nutrient-deficient environment in degradation poplar rhizosphere.

### 2.7. Resistance Genome

**Figure 6:**
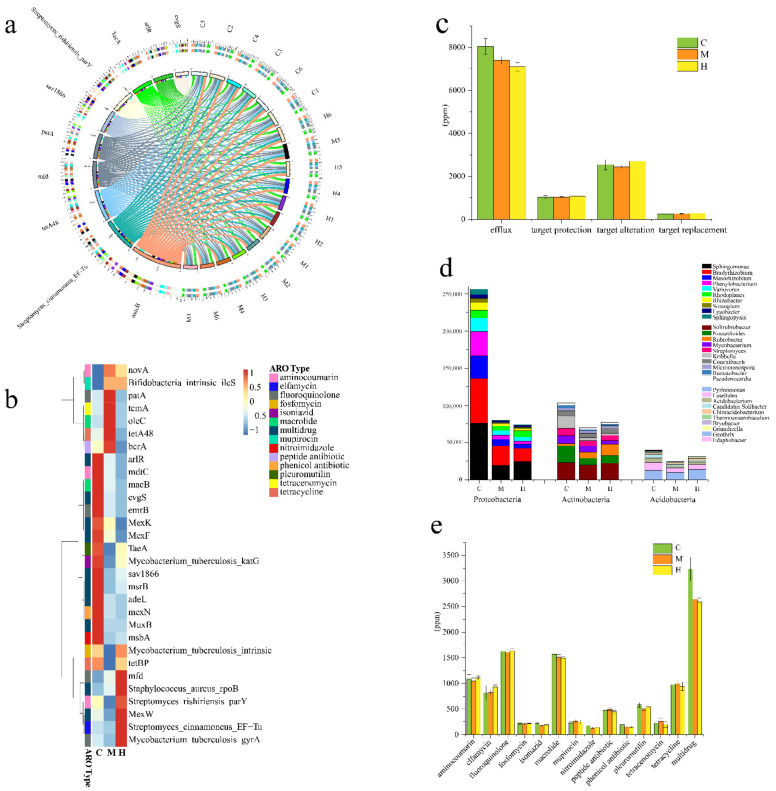
(**a**) The bar width of the bars between ARG types and samples correlates to the percentages of respective ARGs in these samples (in ppm). The different colors in the circle represent different samples and ARGs. (**b**) Heat map analysis of the top 30 most abundant ARGs in at least one group sample using normalized abundance and color-coded based on row z-scores. (**c**) Histogram of four resistance mechanisms of ARGs between groups. (**d**) Histogram of ARGs species attribution analysis. Different colors represent corresponding genera under three phyla. (**e**) Abundance statistics of the top 30 abundant ARGs-related antibiotics.

In order to understand whether ARGs in the rhizosphere of poplars affect its adaptation to the environment and the prevention of pathogen infection, we explored the prevalence of ARGs among different samples. Compared with the group H and M, the group C contained a higher number of resistance genes (Figure S3a). More than 90% of the genes in each sample were not annotated in any ARO, indicating that poplar rhizosphere soil was a very huge drug resistance gene pool with great potential exploitation value. By blasting against the CARD database, there were 576 ARGs in common among a total of 633 ARGs annotated in all samples (Figure S3b). The top 30 abundant ARGs accounted for about 64.4% of all the annotated ARGs and the top 10 abundant resistance genes detected were *macB*, *streptomyces_cinnamoneus_EF-Tu*, *tetA48*, *mfd*, *patA*, and *sav1866*, *Streptomyces_rishiriensis_parY*, *TaeA*, *arlR*, and *evgs*. Macrolides-related ARGs (*macB*) are the most abundant and common in distribution, and the proportion in each sample exceeded 7% (Figure 6a). The ARGs associated with fluoroquinolone, macrolide, tetracycline, aminocoumarin, elfamycin, and pleuromutilin were also found to be in high abundance (Figure 6b,e). Interesting, the abundance of multi-drug resistance genes in all ARGs accounted for the highest proportion, exceeding 26% in this study among all the samples (Figure 6c,e). Moreover, the multidrug resistance genes, including *Evgs*, *adeL*, and *emrB* in group C significantly increased than those in group H and M (Figure 6b). Currently, multidrug resistance genes have been found in various bacteria, such as *Staphylococcus*, *Bacillus*, and *Enterococcus*. Microorganisms tend to develop multidrug resistance, as an economical powerful generation strategy, to overcome environmental pressures. We learned from the local area that no artificial antibiotics on the poplar samples. As a reservoir of ARGs, soil has obvious overlap with clinically resistant genomes, but the factors that affect the composition of ARGs in soil and their movement between genomes and habitats are largely unknown. All detected ARGs mainly provide microbial resistance through four resistance mechanisms: Antibiotic efflux, antibiotic target alteration, antibiotic target protection, and antibiotic target replacement (Figure 6c). The ARGs were assigned to the three dominant phyla, *Proteobacteria*, *Actinobacteria* and *Acidobacteria*, which from the latter two had the same proportions in the three groups, but which from the former in group C (22%) had higher proportions than in group M (20%) and H (19%) (Figure S4). Moreover, the abundance of ARGs from *Sphingomonas*, *Bradyrhizobium*, *Mesorhizobium*, *Phenylobacterium*, *Variovorax*, *Rhizobacter,* and *Sphingopyxis* belonging to *Proteobacteria*, *Nocardioides* and *Kribbella* belonging to *Actinobacteria*, and *Luteitalea* belonging to *Acidobacteria*, were significant higher in group C than those in other two groups. However, the abundance of ARGs from *Rubrobacter* belonging to *Actinobacteria* were increased from C, M to H (Figure 6d). It was previously reported that *Actinobacteria* and *Proteobacteria* were the most abundant potential hosts of multi-drug resistance genes [27], which is also confirmed by our results.

According to statistics based on the abundance of ARGs, antibiotic efflux, antibiotic target alteration and antibiotic target protection are the four main mechanisms that confer host microbial resistance [67]. The C group samples contained more abundant resistance genes, especially more abundant multidrug resistance genes, conferring resistance to more different drug classes, which were directly related to the higher abundance of the microbial community.

## 3. Materials and Methods

### 3.1. Sampling Sites and Sample Collection

Soil samples were collected in 2018 during the early August from the poplar-dominated shelterbelts in Zhangbei County, Hebei Province, China, which is a mid-temperate continental monsoon climate with average temperatures and annual precipitation of 12.6 °C and 410 mm, respectively. The three representative plots (20 m × 20 m), which were undegenerated (C, 114.871, 142.00° E, 41.339, 331.99° N), medium-level degenerated (M, 114.872, 047.84° E, 41.335, 539.52° N) and high-level degenerated plot (H, 114.867, 196.47° E, 41.334, 461.14° N), were defined based on the different degeneration degrees (Figure 1). High-level degradation means the trees lost vitality, the leaves fell off and the branches dried entirely, the root systems cannot get any nutrient from the soil (Figure 1a-H). Medium-level degradation means the trees lost a part of vitality, the treetops were dried entirely but the trunk base still alive, there are branches and leaves in the trunk base (Figure 1a-M). Undegradation means the trees live healthy and less dried from the branches or treetops (Figure 1a-C). Six poplar trees were randomly selected from each plot, and the rhizosphere soils were collected at a depth of 10–30 cm using the five-point sampling method. Soils of the same tree were thoroughly mixed as one sample, sieved through 425 μm mesh, placed in a sterilized bag, immediately placed on ice, and transported to the laboratory, where it was stored at −80 °C until physicochemical parameters measurement and DNA extraction.

### 3.2. Degeneration Data Determination and Soil Characterization

In order to better describe the growth of poplars, diameter at breast height (DBH), tree height, degenerated portion height, and canopy density of all poplars in medium-degenerated (M) and undegenerated (C) quadrat were measured. The physicochemical parameters of the soil were measured as follows. Soil pH was determined using a glass electrode meter (Sartorius, Göttingen, Niedersachsen, Germany) in a suspension of 10 g of soil in 25 mL of distilled water. Soil available phosphorus (AP) was extracted using sodium bicarbonate and then measured by the molybdenum blue method. Soil available potassium (AK) was determined by flame photometry [68,69]. Available nitrogen (AN) was determined by potassium persulfate oxidation. Organic matter (OM) content was determined as described by Walkey and Black [70,71]. Soil total potassium (TK), total phosphorus (TP), total nitrogen (TN), microbiological biomass nitrogen (MBN), and microbiological biomass carbon (MBC) were determined analyzed as previously described [72].

### 3.3. DNA Extraction, Shotgun Sequencing, and Metagenome Assembly

DNA from the soil samples was extracted using the PowerSoil DNA Isolation kit (Cellgeno, Beijing, China) following the manufacturer’s instructions. DNA quality was monitored on 1% agarose gels and concentration was measured using Qubit^®^ dsDNA Assay Kit in Qubit^®^ 2.0 Flurometer (Life Technologies, Carlsbad, CA, USA). Sequencing libraries were generated using NEBNext^®^ Ultra™ DNA Library Prep Kit for Illumina (NEB, Ipswich, MA, USA) according to the manufacturer’s recommendations and index codes were added to attribute sequences to each sample [73]. The library preparation was then sequenced on IlluminaHiSeq PE150 platform at Novogene Company (Beijing, China) with paired-end reads generated. Raw sequencing data was preprocessed to remove low-quality and the host-originated reads to acquire the clean data for subsequent analysis. The clean data has been uploaded into the Sequence Read Archive of NCBI under BioProject PRJNA563959 with the accession number SRP241077.

Clean data of 18 samples were assembled by MEGAHIT (EBI-metagenomic, https://github.com/voutcn/megahit, accessed on 20 October 2018) with parameters“-presets meta-large (—min-count 2—k-min 27—k-max 87—k-step 10)” [74] to generate scaffolds. Assembled scaffolds were broken from N connection and obtained the scaftigs which were then filtered the fragment shorter than 500 bp for statistical analysis [75].

### 3.4. Taxonomic Profiling

Scaftigs (≥500 bp) from each sample were used for open reading frame (ORF) prediction [76,77] through MetaGeneMark (http://exon.gatech.edu/meta_gmhmmp.cgi, accessed on 5 November 2018). For ORF (≤100 nt) predicted, CD-HIT software (https://github.com/weizhongli/cdhit, accessed on 10 November 2018) was adopted to remove redundancy and obtain the unique initial gene catalogue. The clean reads from each sample successfully mapped to the gene catalogue, and the genes that contain reads ≤2 in all 18 soil samples were filtered to obtain non-redundant gene catalogue (unigenes) for further analysis. Based on the number of mapped reads and the length of gene, the abundance information of genes in each sample were counted.

To generate the taxonomic information of the unigenes, DIAMOND software (downloaded from https://github.com/bbuchfink/diamond, accessed on 15 December 2018) was taken to blast the unigenes to the sequences of bacteria, archaea, fungi and virus extracted from the NCBI microNR database (blastp, e-value ≤ 1 × 10^−5^) [78]. Based on the lowest common ancestor (LCA) algorithm implemented in MEGAN software (downloaded from https://www.wsi.uni-tuebingen.de/lehrstuehle/algorithms-in-bioinformatics/software/megan6/, accessed on 25 December 2018), the taxonomic annotation of the unigenes was assigned and the relative abundances of each taxa was calculated.

### 3.5. Network Construction

Networks were constructed for C, M, and H based on the relative abundance of the top 100 genera, account for about 80% of all annotated genera, respectively. The adjacency matrixes (threshold filtering: Correlation coefficient *r* > 0.7; significant *p* < 0.05) were achieved by using rcorr function (R: Hmisc) based on Spearman correlation [79] and visualized by using GePHI (version 0.9.2, downloaded from https://gephi.org/, accessed on 10 February 2019).

### 3.6. Function Annotations

Functional annotations of unigenes were performed through against the Kyoto Encyclopedia of Genes and Genomes (KEGG) database by DIAMOND (blastp, e-value ≤ 1 × 10^−5^). In brief, according to predefined sets in the KEGG database, the identified KO genes were further annotated into different pathways. The relative abundance of different functional hierarchy and the gene number table of each sample in each taxonomy hierarchy were obtained.

### 3.7. Resistance Gene Annotation

The unigenes were aligned to CARD database (https://card.mcmaster.ca/home, accessed on 30 January 2019) by Resistance Gene Identifier (RGI) built in CARD with the parameter setting blastp, e-value ≤ 1 × 10^−30^ [80]. Based on the aligned result, abundance distribution of the resistance genes in each samples, the species attribution analysis and the resistance mechanism of resistance genes analysis were also conducted.

### 3.8. Data Analysis

Six replicate samples each group were analyzed. Unweighted pair-group method with arithmetic mean (UPGMA) clustering based on Bray-Curtis distances were performed to investigate beta-diversity patterns. Metastats method [81] was applied to perform hypothesis testing on species abundance and *p*-values were adjusted to q-values by false discovery rate (FDR) correction to screen species with significant differences. Canoco (version 5.1, Wageningen University & Research, Wageningen, The Netherlands) was used to analyze the effects of soil physicochemical factors on species with Pearson correlation. Metabolic pathways and resistance gene heat maps were converted to Z values based on relative abundance, and the maps were presented using Origin (version 9.1, OriginLab, Northampton, MA, USA).

## 4. Conclusions

Through our analysis in this paper, the microbiome composition and functionality in rhizosphere of poplar are likely influenced by the health condition of poplar and available potassium (AK) of soil. We identified several microbes, *Bradyrhizobium*, *Sphingomonas*, *Mesorhizobium*, *Nocardioides*, *Variovorax*, *Gemmatimonadetes*, *Rhizobacter*, *Pedosphaera*, *Candidatus Solibacter*, *Acidobacterium*, and *Phenylobacterium*, may serve as useful bio-indicators of soil fertility. The highest abundance of multidrug resistance genes and the four mainly microbial resistance mechanisms (antibiotic efflux, antibiotic target protection, antibiotic target alteration and antibiotic target replacement) in healthy poplar rhizosphere corroborated the relationship between soil fertility and microbial activity. The healthy rhizosphere soil harbored microbes with a higher capacity and had more complex microbial interaction network to promote plant growing and reduce intracellular levels of antibiotics. The relationships among rhizosphere soil microbial communities, soil fertility and the host poplar presented here set the stage for a valuable reference database for protection and management policies of forestry. Therefore, we are striving for the continuous sampling of these as well as additional sites in the Three North Shelterbelt to provide new insight into the interactions between microbial communities and the health conditions of poplar.

## Data Availability

The sequencing data has been uploaded into the Sequence Read Archive of NCBI under BioProject PRJNA563959 with the accession number SRP241077 (https://www.ncbi.nlm.nih.gov/sra/?term=SRP241077, accessed on 20 February 2021).

## Supplementary Materials

Supplementary Materials are available online at https://www.mdpi.com/article/10.3390/ijms22073438/s1.
