# Supplementary material for: A Degeneration Gradient of Poplar Trees Contributes to the Taxonomic, Functional, and Resistome Diversity of Bacterial Communities in Rhizosphere Soils
Source: Int J Mol Sci. 2021 Mar 26;22(7):3438. doi: 10.3390/ijms22073438 (PMC8036350; doi:10.3390/ijms22073438)
Supplement: Supplementary file 1 [file ijms-22-03438-s001.pdf]

Additional information

Table S1. Growth indicators of all poplar trees in medium-degenerated and undegenerated poplar plots (20m×20m).

		Sample ID	DBH (cm)	average DBH(cm)	tree height (m)	average tree height(m)	degenerated portion height(m)	average degenerated height(m)	canopy density
	1	M6	22.4		11.7		5.8		
	2		24.3		13.3		4.6		
	3		16.3		10.6		5.3		
	4		16.6		16.7		10.8		
	5		21.2		11.5		11.5		
	6		9.3		8.4		8.4		
	7		18.9		13.6		8.4		
	8		19.5		11.6		5.8		
	9	M5	22		12.4		7		
	10		22.9		11.6		8		
medium-degenerated (20m×20m)	11	M4	23.5	18.30	12.8	11.56	6.7	6.82	0.54
	12		23.5		13		7.2		
	13		11.8		9.6		0.7		
	14		21		11.6		6.4		
	15		11.7		11.5		7.2		
	16	M3	19.1		12.4		7.2		
	17		18.6		12.5		7.3		
	18		14.9		10.1		10.1		
	19		14.3		8.5		5.1		
	20		17.9		11.4		11.4		
	21	M1	21.4		11.2		6.1		

	22		19.3		11.5		8.4		
	23	M2	19.1		12		6.1		
	24		18.9		11.7		11.7		
	25		14.6		8.1		0		
	26		12.7		11.2		0		
	1		29.6		8.1				
	2		25.7		18.7				
	3	C6	16.9		12				
	4		26.2		19.1				
	5		27.9		17.9				
	6		18.1		15.7				
	7		21.8		19.6				
	8		12.3		11.4				
	9		14.2		12.6				
	10	C1	21.1		19.1				
undegenerated poplar (20m×20m)	11		14.6	21.49	10.6	15.46			0.83
	12		39.4		18.6				
	13		14.3		16.1				
	14	C5	35.6		20.7				
	15		17.5		11.4				
	16		18.4		14.1				
	17		22.8		18.1				
	18		26.5		18.4				
	19	C2	16.7		16.4				
	20		25.3		19.8				
	21		20.4		15.9				
	22	C4	13.6		15.2				

	23		23.6		14.2				
	24		30.6		18				
	25		21.4		15.9				
	26	C3	15.6		14.9				
	27		15.4		8.6				
	28		16.3		11.8				

Table S2. The coverage of other plant species in each 20m×20m plots.

		total coverage	average coverage
M (20m×20m)	1m×1m - 1	65%	71.8%
	1m×1m - 2	58%	
	1m×1m - 3	72%	
	1m×1m - 4	83%	
	1m×1m - 5	81%	
H (20m×20m)	1m×1m - 1	78%	79.4%
	1m×1m - 2	75%	
	1m×1m - 3	62%	
	1m×1m - 4	90%	
	1m×1m - 5	92%	
C (20m×20m)	1m×1m - 1	21%	17.2%
	1m×1m - 2	11%	
	1m×1m - 3	18%	
	1m×1m - 4	14%	
	1m×1m - 5	22%	

Note: In each 20m×20m sample plot, five 1m×1m quadrats were taken diagonally to measure the species abundance.

Table S3. Statistical table of sequencing data.

	RawData	CleanData	Scaftigs total length (bp)	Scaftigs Numbers	Scaftigs N50 length (bp)	Scaftigs N90 length (bp)	ORFs number	Integrity :all	Integrity :start	Integrity :end	Integrity :none	ORFs total length (Mbp)	GC percent (%)
C1	15,223.38	15,213.14	645,598,969	807,370	742	530	1,144,201	200,009(17.48%)	407,997(35.66%)	297,582(26.01%)	238,613(20.85%)	579.88	65.02
C2	12,593.86	12,586.28	385,821,231	514,734	693	524	709,226	109,648(15.46%)	257,147(36.26%)	182,083(25.67%)	160,348(22.61%)	348.81	64.31
C3	12,513.15	12,500.32	369,476,711	516,181	671	522	692,362	95,732(13.83%)	253,247(36.58%)	178,817(25.83%)	164,566(23.77%)	332.95	64.13
C4	12,151.01	12,135.39	429,743,026	552,120	727	528	776,996	129,427(16.66%)	280,583(36.11%)	203,196(26.15%)	163,790(21.08%)	386.91	64.44
C5	13,415.32	13,404.45	526,135,752	612,496	785	532	901,263	184,494(20.47%)	310,913(34.5%)	228,019(25.3%)	177,837(19.73%)	469.85	63.72
C6	12,938.51	12,928.23	737,551,553	817,863	850	539	1,256,963	280,256(22.3%)	433,911(34.52%)	323,529(25.74%)	219,267(17.44%)	659.77	64.3
M1	13,068.07	13,058.31	382,351,464	542,827	669	522	709,445	87,960(12.4%)	259,815(36.62%)	182,461(25.72%)	179,209(25.26%)	334.74	64.35
M2	13,681.35	13,619.44	456,913,381	635,865	683	524	683,282	93,230(13.64%)	247,593(36.24%)	178,464(26.12%)	163,995(24%)	320.86	65.07
M3	12,241.55	12,184.79	436,054,792	595,080	694	525	703,140	84,002(11.95%)	255,934(36.4%)	181,140(25.76%)	182,064(25.89%)	331.92	64.4
M4	12,518.56	12,435.58	347,339,906	508,082	651	521	694,918	87,043(12.53%)	255,316(36.74%)	180,580(25.99%)	171,979(24.75%)	324.79	65.79
M5	12,956.74	12,898.42	407,684,551	572,895	674	523	530,444	67,742(12.77%)	192,915(36.37%)	135,163(25.48%)	134,624(25.38%)	251.68	65.56
M6	13,059.63	13,000.30	417,198,226	586,587	672	523	640,024	79,162(12.37%)	235,282(36.76%)	165,536(25.86%)	160,044(25.01%)	300.1	65.03

H1	13,767.52	13,757.42	371,046,289	543,176	649	520	717,618	93,641(13.05%)	263,185(36.67%)	188,062(26.21%)	172,730(24.07%)	342.85	64.75
H2	12,324.57	12,316.55	361,924,858	520,615	659	522	855,276	115,022(13.45%)	314,284(36.75%)	225,574(26.37%)	200,396(23.43%)	412.7	64.51
H3	13,018.62	13,008.08	367,757,941	542,060	646	520	804,982	113,978(14.16%)	294,193(36.55%)	213,322(26.5%)	183,489(22.79%)	391.31	64.42
H4	13,622.26	13,611.25	359,761,869	526,407	649	520	665,476	81,504(12.25%)	243,921(36.65%)	174,177(26.17%)	165,874(24.93%)	312.16	65.31
H5	12,104.10	12,051.07	279,978,503	405,784	649	520	770,201	104,596(13.58%)	283,108(36.76%)	203,518(26.42%)	178,979(23.24%)	365.67	65.54
H6	13,366.75	13,358.29	332,526,501	488,558	646	520	795,853	114,260(14.36%)	292,634(36.77%)	211,452(26.57%)	177,507(22.3%)	371.9	66.95

Table S4. The attribute value of a co-occurring network.

	Total nodes	Total links	positive links	negative links	Average degree (avgK)	Average clustering coefficient (avgCC)	Network density	Modularity
**C**	100	1042	738	304	10.42	0.584	0.105	0.993
**M**	94	1134	1112	22	12.06	0.723	0.13	0.468
**H**	91	324	279	45	7.12	0.587	0.079	0.646

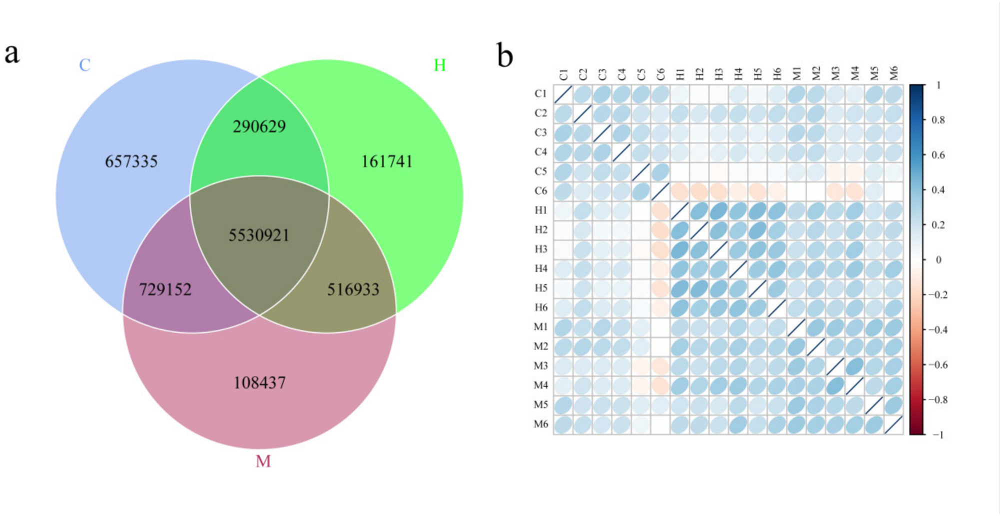

Figure S1. **a** Venn_flower analysis. The overlap represents the number of genes shared by two different treatments, and the others represent the number of exclusive genes in each treatment. **b** Correlation analysis among all samples. The value in the legend represents Spearman's rank correlation coefficient calculated from the normalized abundance of each gene,with a value near 1 indicating 100% similarity between different groups.

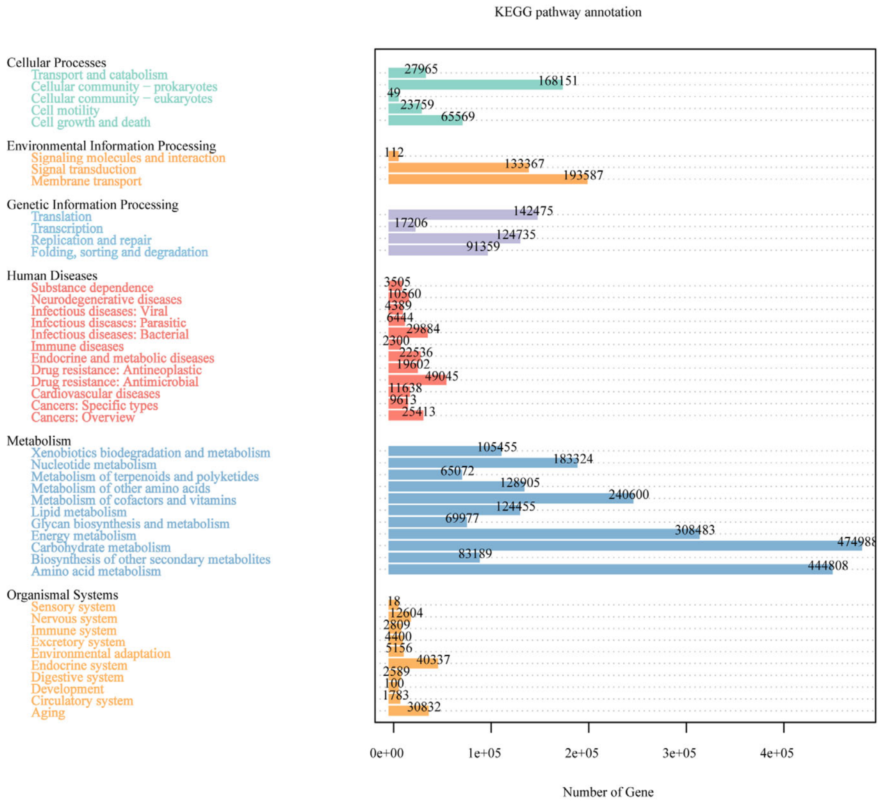

Figure S2. Statistical map of annotated gene number drawn from unigenes annotation results

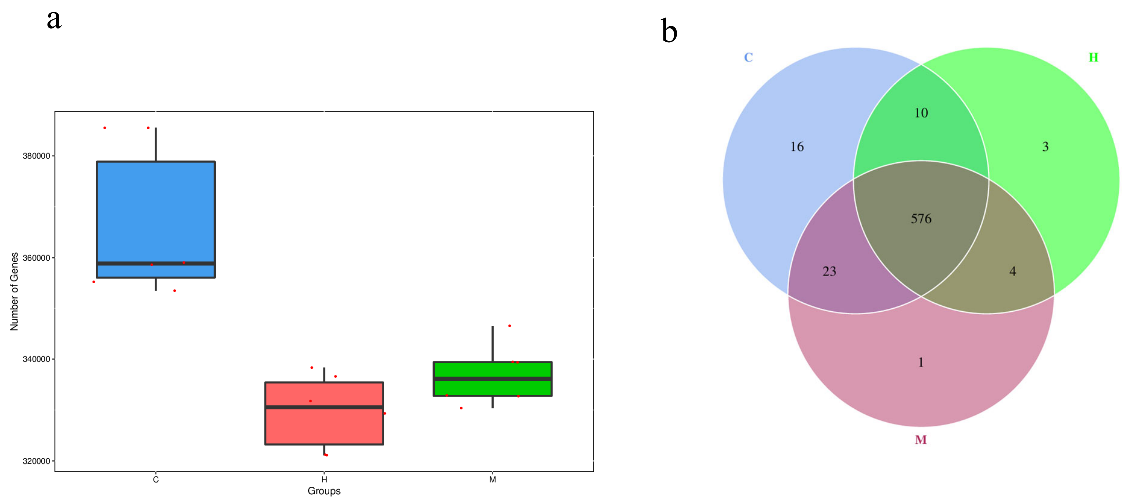

Figure S3. **a** represents the difference in the number of resistance genes among each group; **b** represents the difference in the number of AROs among each group

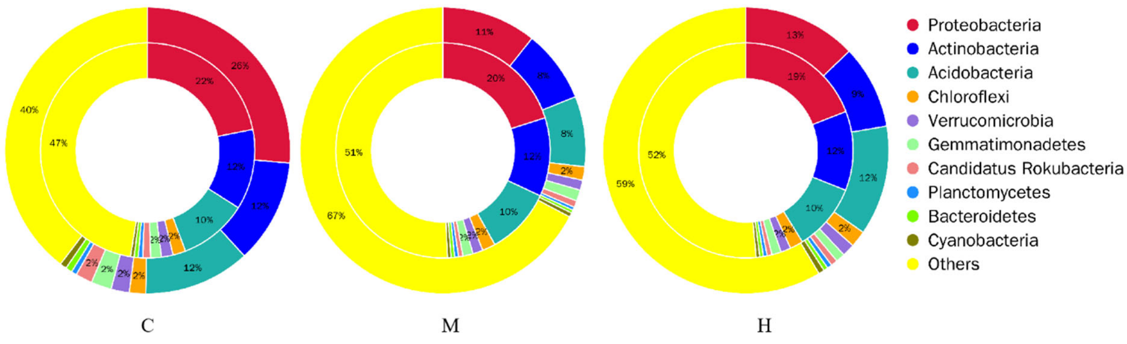

Figure S4. The attribution analysis of ARGs and bacteria species in the samples of C, M and H showed by circle map. The inner circle is the distribution of bacteria species of the total ARGs, and the outer circle is the species distribution of all samples.
